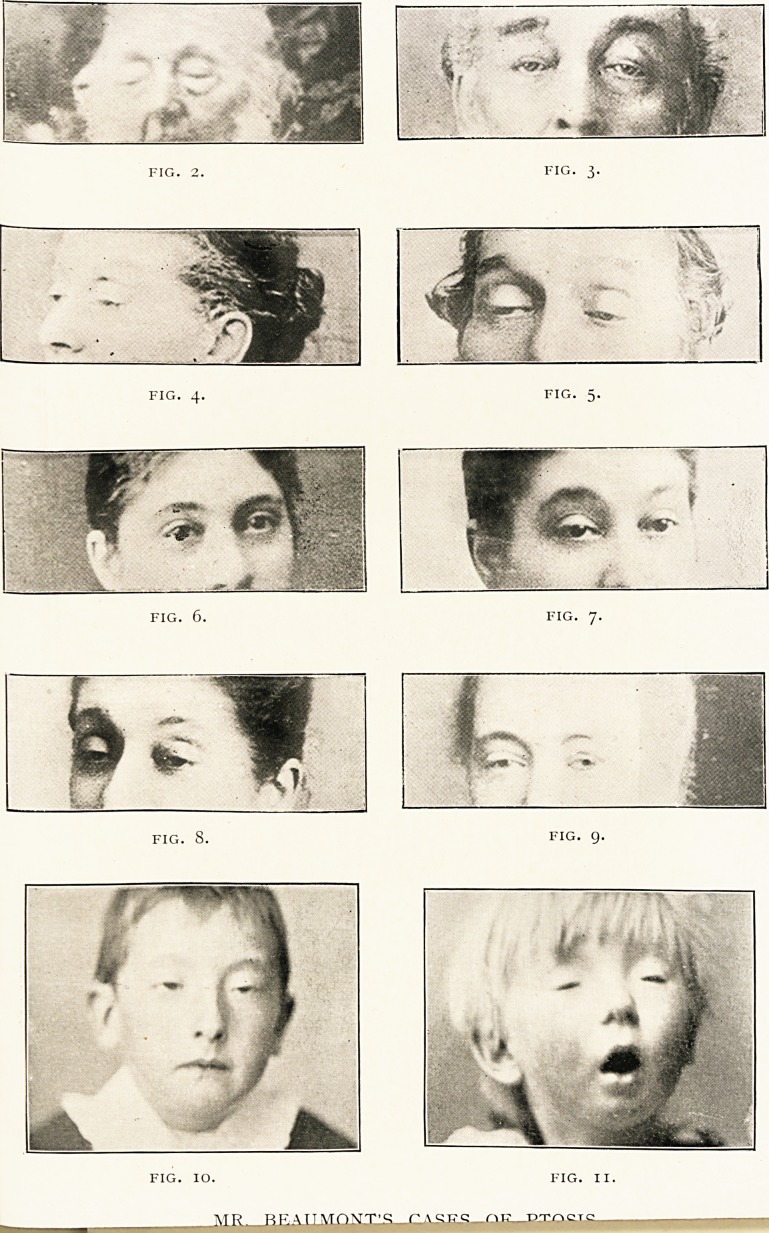# Some Cases of Ptosis

**Published:** 1902-06

**Authors:** W. M. Beaumont

**Affiliations:** Surgeon to the Bath Eye Infirmary


### SOME CASES OF PTOSIS.

BY

W. M. Beaumont, M.R.C.S., L.S.A.,

*Surgeon to the Bath Eye Infirmary.*

---

At a recent meeting of the Bath and Bristol Branch of the British Medical Association[1] I showed some lantern slides illustrating ptosis, and it has been suggested to me that a short *resumé* of the cases might be of interest to the readers of the *Bristol Medico-Chirurgical Journal.*

The ptosis was symptomatic of ophthalmoplegia externa, and the unusual point was that it came on in middle life in twelve members of one family. Ophthalmoplegia externa is not a disease of everyday occurrence, and it may be useful to outline briefly some of its features. In the first place, in a well-marked case, the external ocular muscles are completely paralysed, and the eyes are as immovably fixed in the orbits as they would be if embedded in cement. Patients with complete ophthalmoplegia externa can neither move their eyes upwards, downwards, inwards nor outwards; but there is no paralysis of the internal muscles. The iris is untouched, and accommodation, irritation, and light reflexes are alike active.

The muscles affected are the recti and the obliqui, the levator palpebræ and the orbicularis. The paralysis of the orbicularis is of interest from the fact that formerly this muscle was supposed to get its nerve supply from the facial. Mendel has shown the probability that it comes, *viâ* the facial, from the third nerve nucleus.

Ophthalmoplegia externa may be congenital, or it may come on late in life. It is sometimes associated with syphilis, with locomotor ataxy, or with general paralysis; but sometimes it is not apparently associated with any other disease. It is

[1] November 27th, 1901.

due to a slow degenerative process in the nuclei, but why this degenerative process should occur we do not know.

One fact is very clear, namely, that the muscles which move the eyes are not necessaries to the modern civilised man. He who has neither recti nor obliqui is at no disadvantage in the up-to-date struggle for life. If any justification for this statement were necessary, I would point to the fact that patients with ophthalmoplegia externa may possibly complain of the ptosis, but otherwise they are not usually aware that there is anything wrong with their ocular muscles. It is hardly more difficult to move the head than it is to move the eyes, and the time involved is inappreciable. For all the ordinary duties of life, he whose field of fixation is no greater than his field of vision is the equal of him who has a normal field of fixation. In a case of this disease which I published in the volume of *Brain* for 1890 the disease had probably existed for thirty or forty years, and the only complaint the patient made was of the ptosis. She could accommodate perfectly, and reading, writing, and sewing were as easy to her as they are to ordinary people.

One can well understand in early days that when prehistoric man lived by his muscles, rather than by his wits, rapid movements of the eyes were essential in order for him to avoid his foe or to see his quarry. The anthropoid with the quickest movements would probably survive in the struggle for existence.

In the following series of cases of ophthalmoplegia externa which I have already published[1] the ptosis was well marked; yet, apart from the ptosis, no one of the patients was aware of the disease. In the accompanying chart, reprinted from the Ophthalmological Society's Reports, the male members of the family are represented by squares and the female by circles. The pedigree shows that of the children of the first member affected, as far as is known, three out of five were ophthalmoplegic; in the third generation, five out of eleven; and in the fourth, three out of forty-four. But as the last generation consists mostly of children, and as the abnormality

[1] *U. Kingdom Tr. Ophth. Soc.*, 1900, xx. 258.

Fig. 1.

PEDIGREE.

*a*. Eleven children, sex unknown.
*b*. Sixteen children, sex unknown.

Male □
Female ○

does not develop until maturity is reached, it is possible that other members may be affected later on. That the ptosis is a prominent symptom can be seen from the accompanying diagrams.

In Fig. 2, corresponding to No. 3 in the pedigree, the ptosis appears to be so complete that one wonders that he could see at all.

Fig. 3 (Case 7) shows a male member of the third generation after the condition had been relieved to some extent by operation.

Fig. 4 (Case 8) is a sister of the last.

Fig. 5 (Case 9) is a brother of the last two.

Figs. 6, 7, and 8 (Case 9) represent a female member of the fourth generation. Fig. 6 shows the patient at the age of 23, in the year 1884, before the onset of the ophthalmoplegia. Fig. 7 is the same patient six years later, when the ptosis is seen to be beginning; and in Fig. 8, nine years later, the drooping is still more marked. The patient has recently been relieved by a Pagenstecher operation, but I regret that I am unable to show the very satisfactory result.

Fig. 9 represents Case 12. She lives in Australia, and, except the fact told us by the photograph, I know nothing.

The sleepy expression, so characteristic of ptosis, is well exemplified in Figs. 4, 5, and 8. Fig. 10 shows it also. This patient is not a member of the family, but the case is of interest from the fact that although the child frequently "cried," he was never known to shed a tear, and yet there was no evidence of absence of the lachrymal glands. That babies do not shed tears is well known; but that the onset of the faculty should be delayed beyond seven years is, I think, very exceptional.

It is not my intention to enter into the question of treatment here, and I will only mention that in most of the cases requiring operation that of Pagenstecher was selected; but in the latter ones the procedure of Hess was adopted—in all cases with satisfactory results.

Cases of ptosis are sometimes associated with movements of the muscles of the jaw; but they only occur, so far as I

FIG. 2.

FIG. 3.

FIG. 4.

FIG. 5.

FIG. 6.

FIG. 7.

FIG. 8.

FIG. 9.

FIG. 10.

FIG. 11.

MR. BEAUMONT'S CASES OF PTOSIS.

know, in the congenital cases, and never in those in which the ophthalmoplegia develops later in life. They are decidedly rare, and for this reason it may be of interest to refer to a case which I published in the *Lancet*[1] nine years ago.

The child, S. M., was brought to the Bath Eye Infirmary suffering from epicanthus and ptosis. "There was no visible action of the levators, and the upper and lower eyelids were separated from each other by a mere chink, between which the corneæ could scarcely be seen. When the child wished to use his eyes he assumed the usual attitude adopted in such cases — throwing his head back and looking under the immovable upper lids. But what caused even more distress to the parents than the ptosis was the fact that when their child attempted to use his eyes he invariably opened his mouth. The idiotic expression which this produced was very marked." Fig. 11 shows this very well.

## Figures and Tables

**Fig. 1. f1:**
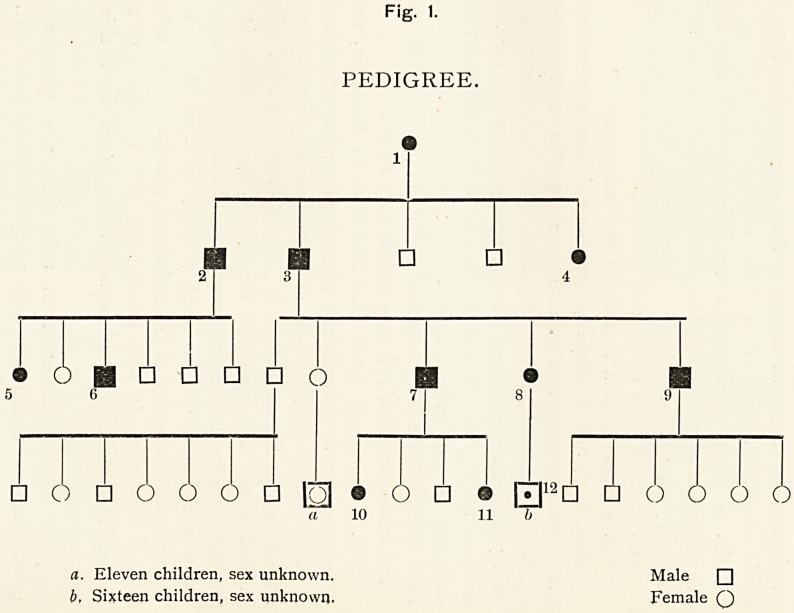


**Fig. 2. Fig. 3. Fig. 4. Fig. 5. Fig. 6. Fig. 7. Fig. 8. Fig. 9. Fig. 10. Fig. 11. f2:**